# Case Report: Cognitive Assessment Before an Amnesic Seizure in Transient Epileptic Amnesia Syndrome

**DOI:** 10.3389/fneur.2022.919706

**Published:** 2022-07-04

**Authors:** Coline Bouyer, Bertrand de Toffol

**Affiliations:** ^1^Service de neurologie Centre Hospitalier de Cayenne, Cayenne, France; ^2^CIC Inserm 1424 CH Cayenne, Cayenne, France

**Keywords:** epilepsy, memory, neuropsychology, transient epileptic amnesia (TEA), amnesic seizure

## Abstract

A patient with transient epileptic amnesia syndrome presented a clinically observable amnesic seizure immediately after a neuropsychological assessment. An hour and a half before the onset of the seizure, the patient progressively developed an isolated alteration of episodic memory. These data question the ictal/interictal distinction in this syndrome as well as the speed of propagation of an epileptic activity.

## Introduction

Transient epileptic amnesia (TEA) is a mesial temporal epileptic syndrome for which the diagnostic criteria, defined by Zeman et al. (1), are: (1) A history of recurrent witnessed episodes of transient amnesia; (2) Cognitive functions other than memory judged to be intact during typical episodes by a reliable witness; (3) Evidence for a diagnosis of epilepsy based on one or more of the following: (a) Epileptiform abnormalities on electroencephalography; (b) The concurrent onset of other clinical features of epilepsy (e.g., lip-smacking, olfactory hallucinations); (c) A clear-cut response to anticonvulsant therapy.

A recent general review (2), based on a substantial patient cohort, concluded that TEA is a distinctive form of late-onset limbic epilepsy. Patients have recurrent episodes of transient amnesia, typically lasting for around 30 min, often when waking, frequently occurring at intervals of around 1 month. Other symptoms can occur, such as olfactory hallucinations (43% of the patients), repetitive questioning (57%) or motor automatisms (41%) (2). During the amnesic attacks, patients remain able to carry on a conversation and act appropriately (3), which suggests the preservation of cognitive functions (other than memory) such as attention, perception, language, and executive functions. Standard interictal neuropsychological assessments (NPA), which evaluate episodic memory on a short temporality, generally show normal performance (3, 4). The majority of patients report interictal memory impairment, specifically an accelerated long-term forgetting (ALF) and autobiographical amnesia (AbA). Thirty % of the patients mention a distinctive form of emotional lability (2). Specific NPA objectify patients' subjective complaint. They evaluate the ALF with a 1 h-delayed recall (5–7), a 24 h-delayed recall (8), a 1 week (or more) delayed recall (4, 9, 10). They test AbA with semi-structured interviews (11, 12). The condition of TEA syndrome is most often of unknown etiology and has a benign prognosis.

We report here the exceptional case of a well-characterized TEA patient who presented an amnesic attack in the immediate aftermath of an NPA. We can thus detail the appearance and progression of subtle cognitive impairments 1 h and a half before the clinical seizure.

## Case Description

We report the case of a man who was 60 years old at the time of the diagnosis. He had been retired for 7 months and was house painter.

The patient came to see a neurologist for recurrent attacks:

- The first one happened four months before his retirement, during a period of overwork. While he was working, he suddenly felt disorientated, didn't know what he was doing.
- Two months later, he had a second attack when waking up. The patient describes the sensation of having a gray veil in front of his face. He felt lost, didn't know what he had to do or where he wanted to go.
- One month later, also while waking up, he had a third attack. Once again, he felt like having a gray veil in front of him and didn't know what he had to do. During the morning, while he was driving, his wife observed that he seemed haggard without any loss of contact or difficulty for driving.
- The next month, when waking up the day after Christmas, the patient felt disorientated and couldn't remember what had happened the day before.
- A fifth episode happened 2 months later, he was shopping with his wife when he felt disorientated and didn't know what he had to do.

Three attacks appeared when the patient was waking up. During the attacks, the patient systematically had a confused look, as if he was lost, iterative questioned the people around him, but he was able to perform motor acts without error. There were no other temporal lobe epilepsy symptoms (such as olfactory hallucinations or motor automatisms) described by the patient or his wife. All these episodes lasted around 5 min with a post critical phase which could last from a few minutes to 1 h. During the post critical phase, the patient had memory impairment which seemed both retrograde—for example, after the acute phase of the first attack, he didn't remember if he had called his client the day before or not—and anterograde — his wife noted that he didn't correctly memorize information at post critical phase but she didn't notice any element of aphasia. The patient could remember that he had had a seizure because he did not feel well but he could not describe what he had done or said. Between the attacks, his wife observed some disturbance of autobiographical memory and an unusual emotionality. The patient had no more complaint.

Brain MRI, TEP-scan, video-EEG, spinal tap and NPA were performed. Clinical seizures were controlled with 100 mg lamotrigine per day.

**Figure 1:**
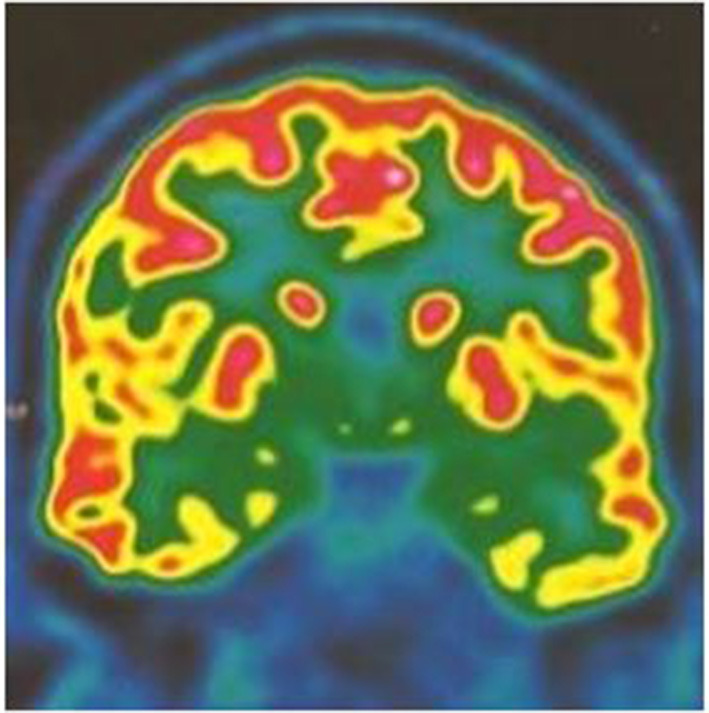
18 FDG PET scan coronal cut showing bilateral mesial temporal hypometabolism more pronounced on the left temporal lobe.

**Figure 2:**
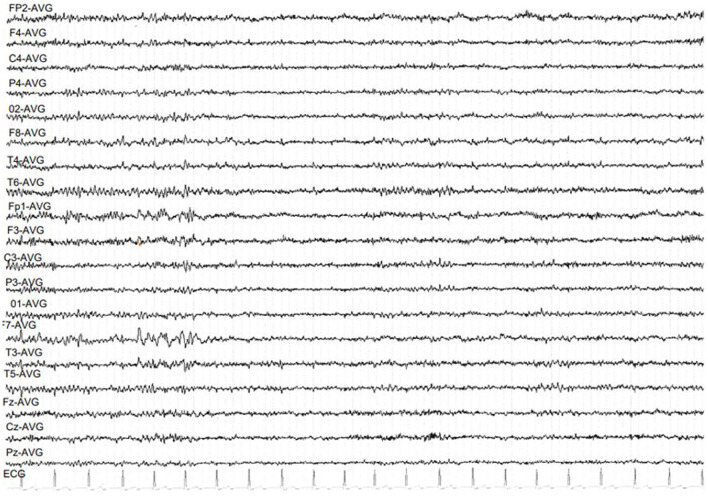
EEG showing slow wave focus associated with spikes on the left temporal lobe.

Brain MRI was normal. PET scan showed bilateral hippocampal and parahippocampal hypometabolism (Figure 1) and video-EEG showed a slow wave focus associated with spikes under the left anterior and middle temporal electrodes especially during sleep (Figure 2). The spinal tap was normal. All known autoimmune antibodies were negative.

## Neuropsychological Assessments

During each NPA, we evaluated the global cognitive efficiency with the MMSE (13). Anterograde verbal memory was evaluated using the logical memory from Weschler Memory Scale III (WMS—III, immediate and 30-min delayed recall, and recognition) (14) and the RL/RI-16 (immediate and 20-min delayed recall) (15). For logical memory subtest, the same two stories were presented to the patient for each NPA but we used three different lists of words to assess the RL/RI-16. These three lists are validated and considered as comparable (15, 16). Anterograde visual memory was assessed with the recall of the Rey-Osterrieth Complex Figure (17), the 10/36 spatial recall test (18) and the faces recognition test from the WMS-III. Verbal working memory was tested with the WAIS-IV digit span subtest (19). Executive functions were tested with Subtests from GREFEX battery (20). Visuoconstruction was assessed with the copy of Rey-Osterrieth Complex Figure. Language was evaluated with the DO 80 denomination test (21). Emotional state was controlled with the HADS (22). In addition, we assessed the Autobiographical memory with the TEMPau task (12, 23) during a second appointment for the first NPA. We also examined long-term consolidation with asking a 1-week delayed recall of the RL/RI-16 list of words to research an ALF. This evaluation is only a clinical observation because there are no norms for this task. We did not ask a 1-week delayed-recall for Logical Memory because of the impaired performance on 20-min delayed recall.

Three NPA were performed (summarized in Table 1). The tests were proposed in the same order during the three NPA (Table 2).

1. First NPA

**Table 1:** Neuropsychological assessments.

		**NPA 1**	**NPA 2**	**NPA 3**	**Cut off**
		**Score**	**Score**	**Score**	
**Mental status**					
MMSE (13)		29/30	28/30	30/30	27
**Memory**					
Working memory					
Digit Span Recall (WAIS IV) (19)	Total	11/19	14/19	13/19	5
Anterograde memory					
- Verbal memory					
RL/RI-16 (15)		List A	List B	List C	
	Immediate Recall	16/16	16/16	16/16	13
	1st Free Recall	6/16	4/16^**^	8/16	center.16
	2^nd^ Free Recall	6/16^*^	7/16	6/16^*^	center.42
	3rd Free Recall	8/16	3/16^**^	10/16	center.93
	1st Total Recall	12/16^*^	8/16^**^	12/16^*^	12
	2^nd^ Total Recall	15/16	9/16^**^	13/16^*^	13
	3rd Total Recall	15/16	11/16^**^	14/16^*^	14
	Recognition	16/16	13/16^*^	16/16	
	20′ delayed Free Recall	9/16	6/16^**^	8/16^*^	8.16
	20′ delayed Total Recall	16/16	10/16^**^	15/16	14
	1 week DFR	0/16		2/16	
	1 week DTR	7/16		5/16	
	FR	0	0	0	
	Intrusions	free recall: 0 induced recall: 2	free recall: 5 induced recall: 14	free recall: 2 Induced recall: 5	
	Intrusions at 1 week	free recall: 0 induced recall: 7		free recall: 2 induced recall: 2	
Logical memory (MEM III) (14)	Immediate recall	7/19	1/19^**^	7/19	5
	Delayed recall	4/19^*^	1/19^**^	3/19^**^	5
	Recognition	22/30 C17-C25	19/30 C3-C9	15/30 ≤C2	C5
- Visual memory					
10/36 (18)	Total immediate recalls	24/30	15/30	22/30	9
	Delayed recall	9/10	3/10^*^	8/10	3
Face recognition (MEM III) (14)	Immediate recognition	9/19			5
	Delayed recognition	10/19			5
Rey complex figure		9/36^*^	7/36^*^	12/36^*^	15
Autobiographical memory					
TEMPau (12, 23)					
Episodic Score	0–17 years old	4			0
	18–30 years old	0^**^			1.69
	Over 30 years old	0^*^			0.15
	5 last years	0^*^			0.19
	12 last months	4			2.29
Global Score	0–17 years old	7^**^			9.09
	18–30 years old	7^**^			9.11
	Over 30 years old	9^*^			8.275
	5 last years	6^**^			7.09
	12 last months	11			9.88
**Language**					
Denomination (21)	DO 80	80/80	79/80	80/80	
**Executive functions**					
**GREFEX Version** **(****20****)**					
Fluency	Animals (2′)	32	25	33	18
	Letter P (2′)	23	26	23	10
Stroop	Interference—Denomination (sec)	95	60	47	90
TMT	B-A (sec)	34	26	17	120
**Visuoconstruction**					
Rey complex figure (17)	Type	I	IV	IV	
	Score	27/36^*^	26/36^*^	30/36	29
**Emotional state**					
HADS (22)	Anxiety	8/21^*^	3/21	11/21^*^	8–10
	Depression	1/21	7/21	4/21	8–10

* *Score below the pathological threshold or below −1.65 standard deviation*;

** *score below −2 standard deviation*.

*NPA, Neuropsychological Assessment; MMSE, Mini Mental State Examination; RL/RI-16, Rappel libre/rappel indicé à 16 items; DFR, Delayed Free Recall; DTR, Delayed Total Recall; FR, False Recognition; C, Centile; TMT, Trail Making Test; HADS, Hospital Anxiety and Depression Scale*.

**Table 2:** Order of tests administration.

**Order**	**Test**	**Estimated duration** **(minutes)**
1.	MMSE	10
2.	RL/RI-16: - Immédiate recall - 1st, 2nd, and 3rd recall - Immediate recognition	25
3.	Rey-osterrieth complex figure: - Copy	3
4.	Digit span subtest (WAIS-IV)	12
5.	Rey-osterrieth complex figure: - Recall	5
6.	RL/RI-16: - 20 min delayed recall	5
7.	Logical memory - Immediate recall	10
8.	10/36: - 1st, 2nd, and 3rd recall	10
9.	TMT	5
10.	Stroop	5
11.	10/36: - Delayed recall	5
12.	Logical memory: - Delayed recall - Recognition	10
13.	Fluency	5
14.	DO 80	5
15.	HADS	10

The first NPA revealed a global preservation of cognitive functions on the standard evaluation, as it has been described in literature except for episodic memory. The RL/RI-16 showed an encoding impairment on the first total recall, and a slight retrieval difficulty but the middle term storage appeared normal. Delayed recall of the logical memory subtest, which requires recalling two stories presented only once, was impaired but delayed recognition remained normal. The patient said that this type of exercise had always been difficult for him. Visual memory appeared efficient except when the test required incident learning.

**Figure 3:**
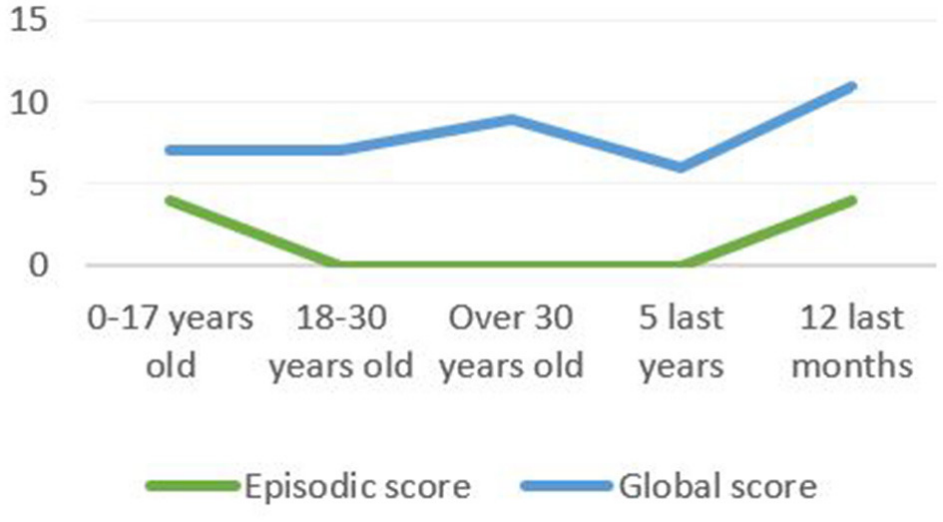
The TEMPau shows a U-distribution with a better performance for childhood episodic memories and for the past year.

Furthermore, according to his complaint, the results suggest an ALF: the patient could not spontaneously recall any item of the RL/RI-16 list of words after 1 week, retrieved seven with help and proposed seven intrusions. The patient also presented an AbA. The impairment was more important for episodic memories which aligns with the literature data. We observed a U-distribution with a better performance for childhood episodic memories and for the past year (Figure 3). The detailed study of the profile showed that the past year episodic memories evoked by the patient concerned the last months and the last days before the evaluation. The patient could not report any episodic memory for the three interim periods (Table 1).

The score of Rey-Orsterrieth Complex Figure was impaired by slight disproportions and one omission but it did not constitute real visuoconstruction impairment.

Emotional state evaluation indicated anxiety manifestations.

In the end, we asked the patient to come again a year later for a new NPA.

2. Second NPA: during an acute phase

As agreed, the patient came 1 year later. He was still taking 100mg of lamotrigine per day. He only had one seizure since the first NPA. It had occurred 2 months before the second NPA, when waking up, and his wife observed the same disorientation and iterative questioning associated with some iterative deglutition movements.

During the interview, the patient was perfectly coherent and gave an informative speech. He did not mention any new complaint. He still reported an unusual emotionality and an AbA. These features did not disturb his daily functioning. There was no autonomy loss and he still managed his rental houses without difficulty. His wife agreed with this information.

The assessment revealed a preservation of global cognitive efficiency. Language, executive functions and working memory were maintained. In contrast, verbal episodic memory was significantly impaired on the RL/RI-16, more than during the first NPA: all the scores were impaired and below two standard deviations. The repetition of immediate recalls, during which the missing items are recalled by the examiner, didn't significantly improve the scores so we could notice an encoding impairment. Furthermore, the patient gave 19 intrusions. Immediate recognition was also impaired. Immediate recall of logical memory subtest was substantially impaired whereas it was normal during the first NPA. Visual episodic memory evaluation objectified an encoding difficulty and a storage impairment of visuospatial information during the 10/36 task. This time visuoconstruction was impaired by deformations and a planning trouble that was not present during the first NPA. However, the HADS did not show anxiety or depression symptoms.

We therefore observed, from this second NPA, an isolated encoding and storage impairment in verbal and visual episodic memory with only an associated visuoconstruction difficulty and a diminution of anxiety.

**Figure 4:**
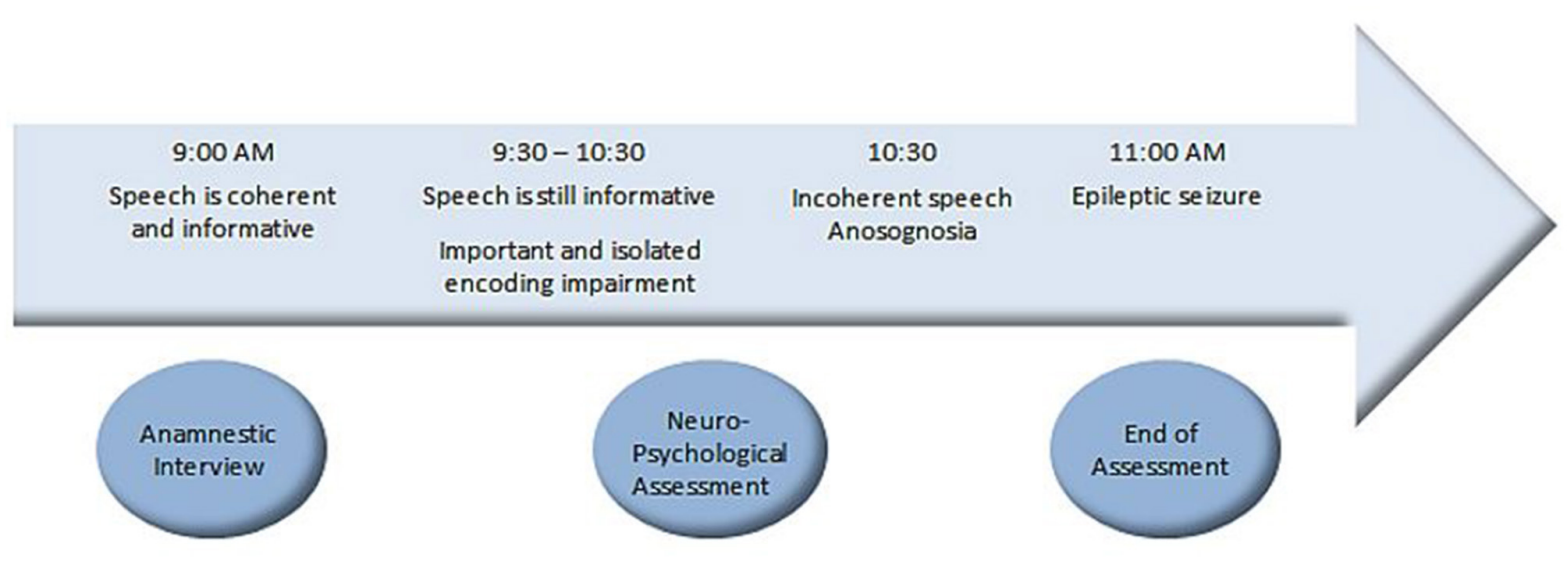
Unfolding facts.

During the last part of the examination, the patient began to justify his results, saying that he had never been good for memory tasks. At the end of the tests assessment, the psychologist took some time to discuss the results. That is when the patient began to give unclear answers during the conversation. He gave repetitive justifications. When the psychologist asked questions about the daily life, he gave irrelevant responses. When the psychologist said that the NPA was complete and that he was going to call his wife, the patient had a clinically observable seizure with pallor of the face, repetitive swallowing movements, disorientation and comprehension disorder. He repeated “I don't understand what you are saying”. He then repeated what the psychologist said by echolalia. The episode lasted 5 min then the patient could speak normally and said that he didn't feel well during the seizure but did not remember that he was repeating the same sentences. Figure 4 sums up the unfolding facts.

Thus, more than an hour and a half before the observation of a clinical seizure, a significant and isolated impairment of verbal and visual episodic memory encoding and storage was measured, followed by some degree of anosognosia (patient attempted to justify the difficulties). Anxiety was reduced on the mood self-assessment questionnaire. Given the occurrence of an amnesic seizure at the end of the second NPA, the patient was asked to come back 1 month later to reassess the tests.

The appointment was set up during the afternoon, to avoid stressing the patient on awakening.

3. Third NPA

When the patient came back 1 month later, he had not had any seizure. His complaint was still the same. He remembered that he came for the NPA 2 one month before and that he had a seizure.

The third NPA revealed a preservation of global cognitive efficiency, language, executive functions and working memory. Visuoconstruction was normalized. The assessment of verbal episodic memory showed an encoding and consolidation difficulty but the 20-min delayed recall showed a normal storage. As on the first NPA, logical memory subtest showed impaired delayed recall, and the recognition was also impaired. Emotional state evaluation indicated anxiety manifestations.

An ALF was observed 1 week later: only two words of the RL/RI-16 list were spontaneously recalled and three more with the indication of the category, with four intrusions.

Overall, the third NPA showed substantially the same clinical profile than during the first NPA (Table 1). One year after the first evaluation, the cognitive profile was stable; the patient was still independent in daily life and managed his finances without any difficulty.

## Comments

This patient with TEA syndrome, characterized by amnesic episodes subjectively controlled by 100 mg lamotrigine, had a normal standard neuropsychological assessment on two occasions (NPA 1 and 3) but showed an accelerated forgetting and autobiographical memory impairment, as described in literature (2). The progressive occurrence of an encoding impairment an hour and a half before a clinical seizure requires reconsideration of the classical ictal/interictal distinction on the one hand and the speed of propagation of an epileptic discharge on the other. A clinically observable seizure is only “the tip of the iceberg”. Subtle cognitive symptoms can set in within several minutes. Our observation shows that, in an apparently asymptomatic subject, there may be a reversible prolonged encoding impairment of epileptic origin. Ten cases of surface EEG recording during an amnesic seizure were reported; 8/10 cases showed bilateral temporal seizure activity, and the others had unilateral temporal seizure activity (one left sided and one right sided). Amnesia was observed as an ictal phenomenon in six cases and as postictal in four cases (24). The precise temporal relationship between seizure onset and offset and the associated amnesia is, however, uncertain in the majority of cases of TEA (2). Epileptiform abnormalities on the EEG are rarely seen on a standard waking EEG but often on a nocturnal prolonged sleep EEG (25). Hippocampal interictal spikes recorded on S-EEG during sleep have been shown to disrupt consolidation due to transfer interference between the hippocampus and neocortex (26). It can be suggested that accelerated forgetting and impaired autobiographical memory are the consequence of insufficiently controlled epileptic activity. Some authors consider that ALF results from a consolidation and/or reconsolidation disorder and is consistent with a mesial temporal dysfunction. Encoding process remains preserved (7, 10). ALF could result from a subclinical epileptic activity, impacting the memory-related brain areas (3), which would disturb a long-term stabilization (27).

Our case report raises an important practical question: Should treatment be given to suppress observable seizures or should treatment be enhanced to reduce interictal memory impairment? Should one try to suppress the spikes seen on the sleep EEG in TEA syndrome?

## Data Availability Statement

The original contributions presented in the study are included in the article/supplementary material, further inquiries can be directed to the corresponding author.

## Ethics Statement

Ethical review and approval was not required for the study on human participants in accordance with the local legislation and institutional requirements. The patients/participants provided their written informed consent to participate in this study.

## Author Contributions

CB performed the neuropsychological assessment and drafted the paper. BT shared his theoretical knowledge and drafted the paper. Both authors contributed to the article and approved the submitted version.

## Conflict of Interest

BT was employed by CIC. The remaining author declares that the research was conducted in the absence of any commercial or financial relationships that could be construed as a potential conflict of interest.

## Publisher's Note

All claims expressed in this article are solely those of the authors and do not necessarily represent those of their affiliated organizations, or those of the publisher, the editors and the reviewers. Any product that may be evaluated in this article, or claim that may be made by its manufacturer, is not guaranteed or endorsed by the publisher.

## References

[1] Zeman AZJ Boniface SJ Hodges JR. Transient epileptic amnesia: a description of the clinical and neuropsychological features in 10 cases and a review of the literature. J Neurol Neurosurg Psychiatry. (1998) 64:435–43. 10.1136/jnnp.64.4.435 9576532 PMC2170058

[2] Baker J Savage S Milton F Butler C Kapur N Hodges J. The syndrome of transient epileptic amnesia: a combined series of 115 cases and literature review. Brain Commun. (2021) 3:fcab038. 10.1093/braincomms/fcab038 33884371 PMC8047097

[3] Butler C Zeman A. Syndromes of Transient Amnesia. In: The Neurology of Conciousness. (Amsterdam, Netherlands: Elsevier) (2016). p. 365–78.

[4] Mosbah A Tramoni E Guedj E Aubert S Daquin G Ceccaldi M. Clinical, neuropsychological, and metabolic characteristics of transient epileptic amnesia syndrome. Epilepsia. (2014) 55:699–706. 10.1111/epi.12565 24580051

[5] Atherton KE Nobre AC Zeman AZ Butler CR. Sleep-dependent memory consolidation and accelerated forgetting. Cortex. (2014) 54:92–105. 10.1016/j.cortex.2014.02.009 24657478 PMC4007033

[6] Hoefeijzers S Dewar M Della Sala S Butler C Zeman A. Accelerated long-term forgetting can become apparent within 3–8 hours of wakefulness in patients with transient epileptic amnesia. Neuropsychology. (2015) 29:117–25. 10.1037/neu0000114 25089646 PMC4296931

[7] Jansari AS Davis K McGibbon T Firminger S Kapur N. When “long-term memory” no longer means “forever”: analysis of accelerated long-term forgetting in a patient with temporal lobe epilepsy. Neuropsychologia. (2010) 48:1707–15. 10.1016/j.neuropsychologia.2010.02.018 20178808

[8] Martin RC Loring D Meador KJ Lee GP Thrash N Arena JG. Impaires long-term retention despite normal verbal learning in patients with temporal lobe dysfunction. Neuropsychology. (1991) 5:3–12. 10.1037/0894-4105.5.1.3

[9] Muhlert N Milton F Butler CR Kapur N Zeman AZ. Accelerated forgetting of real-life events in Transient Epileptic Amnesia. Neuropsychologia. (2010) 48:3235–44. 10.1016/j.neuropsychologia.2010.07.001 20620156

[10] Tramoni E Felician O Barbeau EJ Guedj E Guye M Bartolomei F. Long-term consolidation of declarative memory: insight from temporal lobe epilepsy. Brain. (2011) 134:816–31. 10.1093/brain/awr002 21354976

[11] Kopelman MD Wilson BA Baddeley AD. The autobiographical memory interview: a new assessment of autobiographical and personal semantic memory in amnesic patients. J Clin Exp Neuropsychol. (1989) 11:724–44. 10.1080/01688638908400928 2808661

[12] Piolino P Desgranges B Eustache F. La mémoire autobiographique: théorie et pratique. Marseille: Solal. (2000).

[13] Derouesne C Poitreneau J Hugonot L Kalafat M Dubois B Laurent B. Mini-mental state examination: a useful method for the evaluation of the cognitive status of patients by the clinician. Consensual French version. Presse Medicale Paris Fr 1983. (1999) 28:1141–8. 10399508

[14] Wechsler D. MEM-III, Echelle clinique de mémoire, 3ème édition. ECPA. (2001).

[15] Van der Linden M. L'évaluation des troubles de la mémoire: présentation de quatre tests de mémoire épisodique (avec leur étalonnage). Marseille: SOLAL. (2004).

[16] Stoykova R Matharan F Raoux N Amieva H. An alternative word-list for the Free and cued selective reminding test (FCSRT): list presentation and reliability study. Gériatrie Psychol Neuropsychiatr Viellissement. (2013) 11:317–22. 10.1684/pnv.2013.0416 24026134

[17] Rey A. Manuel du Test de copie et de reproduction de mémoire de figure géométriques complexes. Paris: ECPA (1959).

[18] Bever C Grattan L Panitch H Johnson K. The brief repeatable battery of neuropsychological tests for multiple sclerosis: a preliminary serial study. Mult Scler J. (1995) 1:165–9. 10.1177/135245859500100306 9345448

[19] Wechsler D. WAIS-IV, *Echelle d'Intelligence de Wechsler pour Adultes, 4ème édition*. Paris: ECPA (2011).

[20] Godefroy O. Fonctions exécutives et pathologies neurologiques et psychia: évaluation en pratique clinique. Marseille: Ed Solal (2008).

[21] Deloche G Metz-Lutz MM Kremin H Hannequin D Ferrand L Perrier D. Test de dénomination orale de 80 images : DO 80. Paris: ECPA (1989).

[22] Zigmond AS Snaith RP. The Hospital Anxiety and Depression Scale. Acta Psychiatr Scand. (1983) 67:361–70. 10.1111/j.1600-0447.1983.tb09716.x 6880820

[23] Piolino P. La mémoire autobiographique : théorie et pratique en neuropsychologie. Revue Québécoise de Psychologie. (2006) 3:1–20.

[24] Butler CR Zeman AZ. Recent insights into the impairment of memory in epilepsy: transient epileptic amnesia, accelerated long-term forgetting and remote memory impairment. Brain. (2008) 131:2243–63. 10.1093/brain/awn127 18669495

[25] Ramanan VK Morris KA Graff-Radford J Jones DT Burkholder DB Britton JW. Transient Epileptic Amnesia: A Treatable Cause of Spells Associated With Persistent Cognitive Symptoms. Front Neurol. (2019) 10:939. 10.3389/fneur.2019.00939 31555199 PMC6724577

[26] Lambert I Tramoni-Negre E Lagarde S Roehri N Giusiano B Trebuchon-Da Fonseca A. Hippocampal Interictal Spikes during Sleep Impact Long-Term Memory Consolidation. Ann Neurol. (2020) 87:976–87. 10.1002/ana.25744 32279329

[27] McGaugh JL. Memory—A century of consolidation. Science. (2000) 287:248–51. 10.1126/science.287.5451.248 10634773

